# Bioelectrical and cytoskeletal patterns correlate with altered axial polarity in the follicular epithelium of the *Drosophila* mutant *gurken*

**DOI:** 10.1186/s12861-020-00210-8

**Published:** 2020-03-13

**Authors:** Susanne Katharina Schotthöfer, Johannes Bohrmann

**Affiliations:** RWTH Aachen University, Institut für Biologie II, Abt. Zoologie und Humanbiologie, Worringerweg 3, 52056 Aachen, Germany

**Keywords:** *Drosophila melanogaster*, Oogenesis, Electrochemical gradient, Follicle cell, *Gurken*, Pattern formation

## Abstract

**Background:**

Bioelectrical signals are known to be involved in the generation of cell and tissue polarity as well as in cytoskeletal dynamics. The epithelium of *Drosophila* ovarian follicles is a suitable model system for studying connections between electrochemical gradients, patterns of cytoskeletal elements and axial polarity. By interactions between soma and germline cells, the transforming growth factor-α homolog Gurken (Grk) establishes both the anteroposterior and the dorsoventral axis during oogenesis.

**Results:**

In the follicular epithelium of the wild-type (wt) and the polarity mutant *grk*, we analysed stage-specific gradients of membrane potentials (V_mem_) and intracellular pH (pH_i_) using the potentiometric dye DiBAC_4_(3) and the fluorescent pH-indicator 5-CFDA,AM, respectively. In addition, we compared the cytoskeletal organisation in the follicular epithelium of wt and *grk* using fluorescent phalloidin and an antibody against acetylated α-tubulin. Corresponding to impaired polarity in *grk*, the slope of the anteroposterior V_mem_-gradient in stage S9 is significantly reduced compared to wt*.* Even more striking differences in V_mem_- and pH_i_-patterns become obvious during stage S10B, when the respective dorsoventral gradients are established in wt but not in *grk*. Concurrent with bioelectrical differences, wt and *grk* exhibit differences concerning cytoskeletal patterns in the follicular epithelium. During all vitellogenic stages, basal microfilaments in *grk* are characterised by transversal alignment, while wt-typical condensations in centripetal follicle cells (S9) and in dorsal centripetal follicle cells (S10B) are absent. Moreover, in *grk*, longitudinal alignment of microtubules occurs throughout vitellogenesis in all follicle cells, whereas in wt, microtubules in mainbody and posterior follicle cells exhibit a more cell-autonomous organisation. Therefore, in contrast to wt, the follicular epithelium in *grk* is characterised by missing or shallower electrochemical gradients and by more coordinated transcellular cytoskeletal patterns.

**Conclusions:**

Our results show that bioelectrical polarity and cytoskeletal polarity are closely linked to axial polarity in both wt and *grk*. When primary polarity signals are altered, both bioelectrical and cytoskeletal patterns in the follicular epithelium change. We propose that not only cell-specific levels of V_mem_ and pH_i_, or the polarities of transcellular electrochemical gradients, but also the slopes of these gradients are crucial for cytoskeletal modifications and, thus, for proper development of epithelial polarity.

## Background

**Fig. 1:**
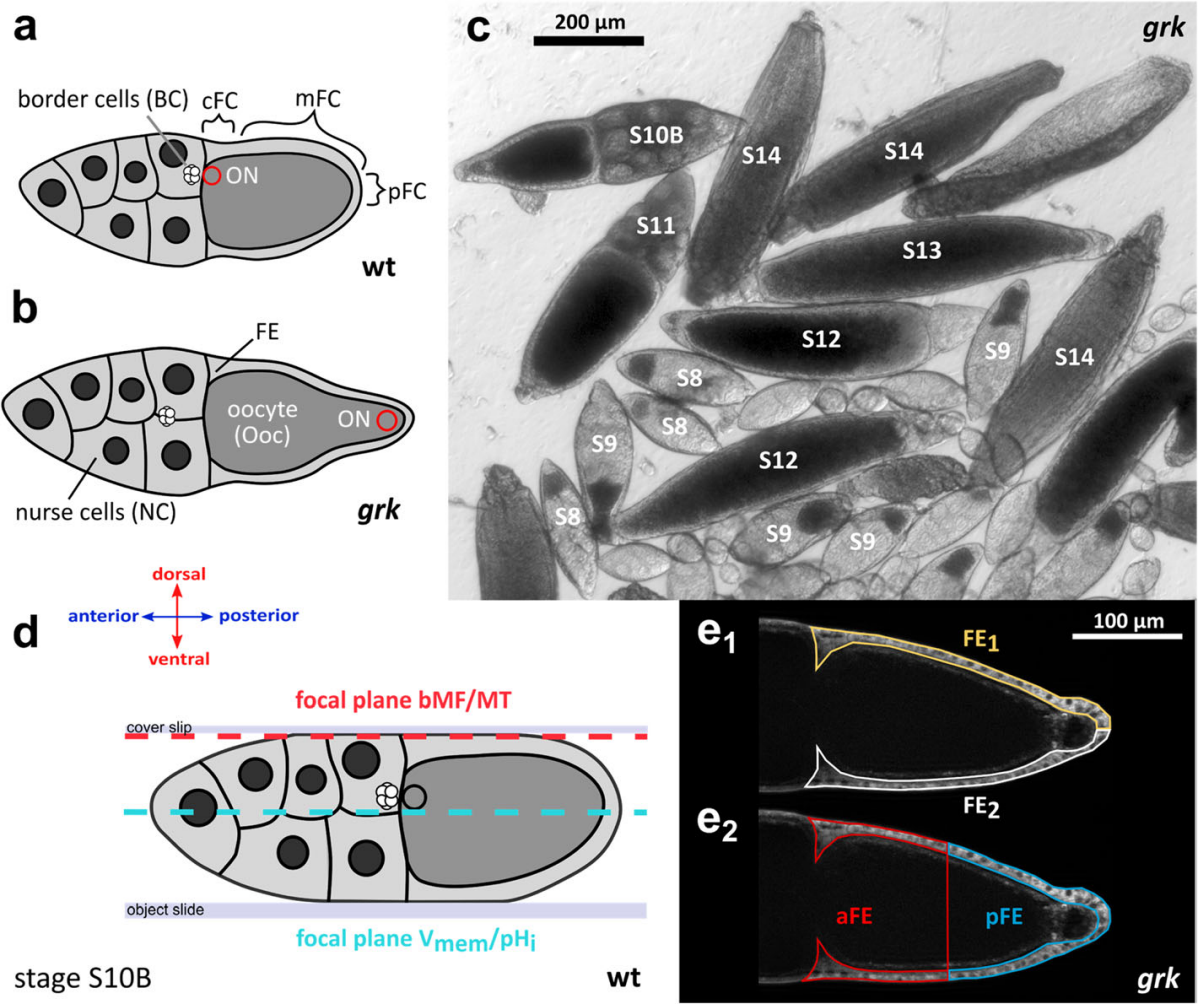
Comparison of wt and *grk* follicles. **a** The dorsal side of wt S10B is defined by a thicker, columnar follicular epithelium (FE) and by an anterodorsal position of the oocyte nucleus (ON, red circle; cFC, centripetal follicle cells; mFC, mainbody follicle cells; pFC, posterior follicle cells). **b***grk* S10B lacks dorsoventral (d-v) polarity and is characterised by a uniform cuboidal, ventralised FE covering the oocyte (Ooc). While, in wt S10B, border cells (BC) are located close to the ON, in *grk* S10B, disrupted body-axis formation leads to undefined positioning of BC amongst the nurse cells (NC). The *grk* ON is often located at the posterior end of the Ooc in a typical protrusion. **c** Transheterozygous combinations of *grk* alleles HF48 and 2B6 result in ventralised *grk* follicles of all vitellogenic stages (S8–14; bright-field image). In S12–14, wt-typical dorsal respiratory appendages are missing and a second micropylar structure appears at the posterior end. **d** To visualise basal microfilaments (bMF) and microtubules (MT) in the FE, tangential optical sections using structured-illumination microscopy (SIM; focal plane: red line) were used. For analysis of V_mem_- and pH_i_-patterns, median optical sections (SIM; focal plane: turquoise line) were used. **e** Quantification of transversal (**e**_**1**_) and anteroposterior (a-p; **e**_**2**_) gradients of V_mem_ and pH_i_, respectively, in the FE of S10B. Example of a *grk* follicle (SIM) where DiBAC-fluorescence intensities of FE_1_ (area marked in yellow) and FE_2_ (white) as well as of aFE (red) and pFE (blue) were measured using ImageJ (“mean grey value”). In wt follicles, the d-v axis was identified via the anterodorsal position of the ON, and the fluorescence intensities of the dorsal and ventral FE were quantified accordingly

Spatiotemporal electrochemical patterns affect cytoskeletal dynamics and play a role in defining spatial coordinates of tissues and organs in several species [1–8]. Therefore, it is tempting to investigate V_mem_- and pH_i_-gradients in relation to cytoskeletal patterns in a *Drosophila* mutant with disturbed axial polarity. Ovarian follicles of the mutant *gurken* (*grk*) show morphological defects concerning both the anteroposterior (a-p) and the dorsoventral (d-v) axis [9, 10]. In *grk* follicles, the oocyte nucleus (ON) is located at the posterior end of the oocyte (Ooc), and the follicular epithelium (FE) has a transversally uniform appearance (Fig. 1). Since ON movement to an anterodorsal position fails to occur in *grk*, both the longitudinal and the transversal axis are not correctly defined [11, 12]. In addition, Grk is required for border-cell (BC) migration to a position adjacent to the ON [13].

Grk, a transforming growth factor-α (TGF-α) homolog, is a ligand of the epidermal growth-factor receptor (EGFR) Torpedo (Top)/DER, and functions as a spatially restricted signal to activate the Egfr-pathway in follicle cells (FC) [14, 15]. Two rounds of Grk-Egfr signalling at different times during oogenesis generate axial polarity. In early oogenesis (stages S6–7), Egfr-activation in posterior FC (pFC) defines a-p polarity, whereas in mid-oogenesis (S9), restriction of Egfr-activity to dorsal FC determines d-v polarity [16]. Localised Grk-Egfr signalling depends on the position of the ON [9, 17, 18]. In strong *grk* mutants, both anterior and posterior FC adopt anterior fates, as indicated by the anterior-specific FC-marker *slbo*, and micropylar structures develop at both poles [9, 17]. In the wild-type (wt), the pFC, in return, signal back to the Ooc, inducing a reorganisation of the microtubule (MT) cytoskeleton and leading to the MT-dependent migration of the ON to an anterior position [12, 19]. As a result, the overlying FC receive the Grk-signal and adopt dorsal fates [16, 20, 21]. This symmetry-breaking step is likely to be a prerequisite for the asymmetrical distribution or activation of ion-transport mechanisms in the FE observed later in development [4].

Participation of bioelectrical signals during axis formation has been demonstrated, e. g., for left-right patterning in *Xenopus* and chick embryos [22, 23], for lateral embryonic eye patterning in *Xenopus* [24], and for a-p patterning in planaria [25, 26]. In particular, the cytoskeleton is an attractive candidate for bioelectrical signalling, since binding of actin-associated factors [27, 28] as well as contractility of actomyosin complexes are controlled by pH_i_ [29]. On the other hand, MT are known to amplify electrical signals [30, 31], and modifications of the cytoskeletal organisation have been shown to be promoted by changes in V_mem_ [8, 32, 33].

For some *Drosophila* mutants with altered axial polarity, connections between morphological polarity and bioelectrical signals have already been described: For example, in *egalitarian* or *Bicaudal-D* mutant follicles, where no Ooc and no a-p or d-v polarity is established, aberrant patterns of extracellular ionic currents correlate with disturbed axial polarity [34–36]. In addition, in follicles of the mutant *dicephalic*, where NC appear at both ends of the Ooc, altered current patterns correlate with impaired a-p polarity [34, 35].

Previous studies on cytoskeletal functions in the FE of *Drosophila* have revealed the requirement of MT in posterior migration of BC (to the Ooc) and in centripetal migration of FC (between NC and Ooc) [37]. On the other hand, the organisation of microfilaments (MF) in the FE corresponds to FC differentiation and plays a decisive role in shaping the follicle along its longitudinal axis [38, 39]. It has also been shown that pH_i_- and V_mem_-changes induced by several inhibitors of ion-transport mechanisms located in the FE [7] simulate naturally occurring bioelectrical changes [4] and lead to alterations of MF- and MT-patterns as observed during FC differentiation [8]. Therefore, gradual modifications of electrochemical signals can serve as physiological means to regulate cell and tissue architecture by modifying cytoskeletal patterns [8].

In the present study, we compare wt and *grk* follicles with regard to their bioelectrical signals, using a fluorescent pH-indicator and a potentiometric dye [4, 7]. In addition, we compare wt and *grk* follicles with regard to their cytoskeletal organisation, using fluorescent phalloidin and an antibody against acetylated α-tubulin [8]. Since, in the wt FE, changes in cytoskeletal patterns are linked to changes in bioelectrical properties, it is tempting to analyse correlations between bioelectrical polarity, cytoskeletal polarity and axial polarity in the polarity mutant *grk*.

## Results

### Bioelectrical differences between wt and *grk*

**Fig. 2:**
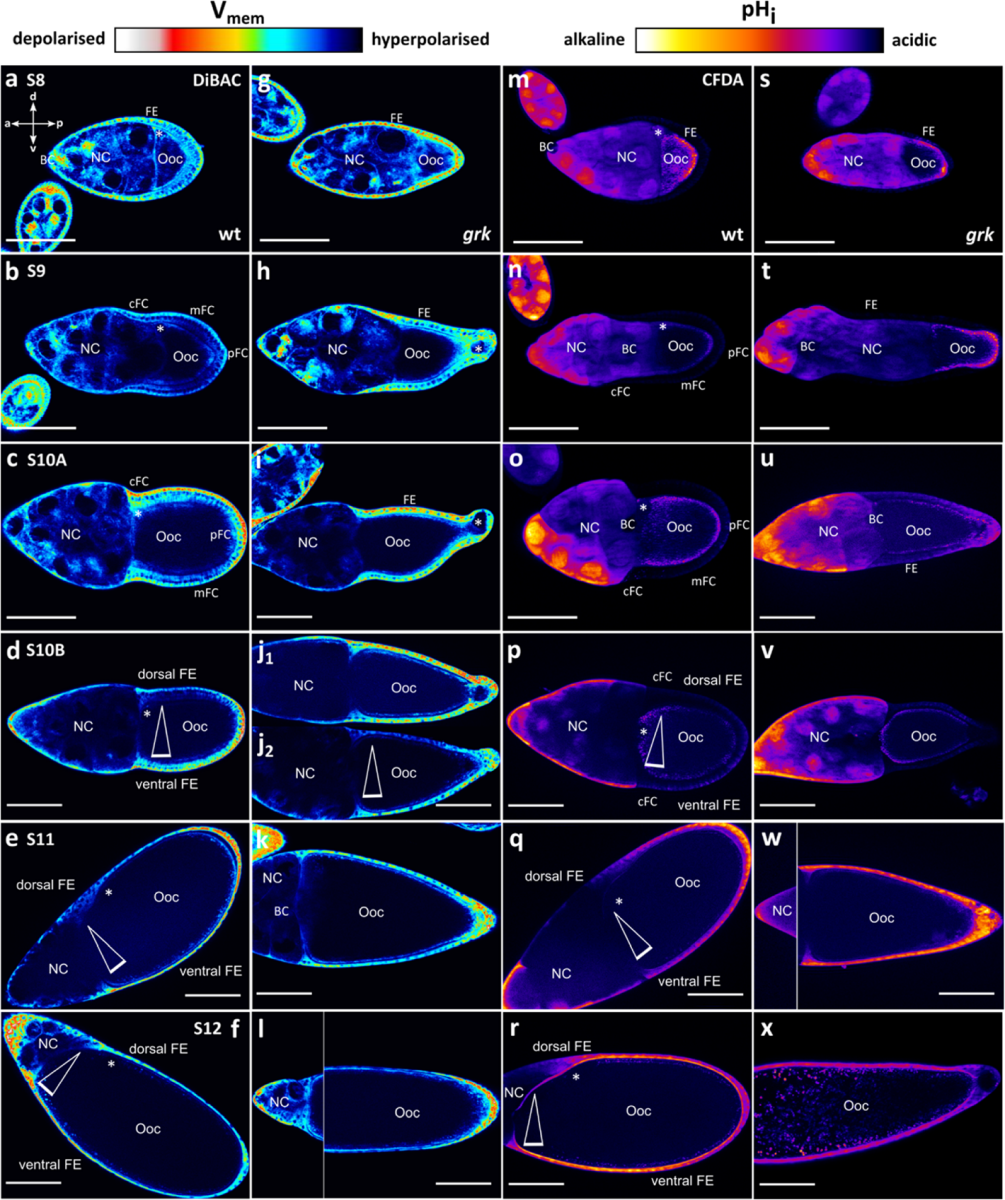
Typical dorsoventral electrochemical gradients, as observed in the wt FE beginning with S10B, are missing in *grk*. DiBAC staining (V_mem_, **a-l**) and CFDA staining (pH_i_, **m-x**); median optical sections (SIM) of typical S8–12 follicles. **a-l** Pseudocolor images of DiBAC stained wt (**a-f**) and *grk* (**g-l**) follicles. Relative depolarisation of V_mem_ is indicated by stronger (red), relative hyperpolarisation by weaker (blue) fluorescence intensities (scale bars represent 100 μm; composed pictures show different regions of the same follicle; positions of the ON are marked with asterisks). **m-x** Pseudocolor images of CFDA stained wt (**m-r**) and *grk* (**s-x**) follicles. Relatively alkaline pH_i_ is indicated by stronger (yellow), relatively acidic pH_i_ by weaker (blue) fluorescence intensities. In early to mid-vitellogenic S8-10A (**a-c, g-i, m-o, s-u**), a-p gradients, but no d-v gradients, of both V_mem_ and pH_i_ are detectable in the FE of both wt and *grk*. Compared to wt, the mFC in *grk* are less hyperpolarised relative to cFC, and the whole FE is more depolarised. More striking differences between wt and *grk* become obvious during S10B when d-v gradients (triangles indicate fluorescence-intensity gradients) of both V_mem_ and pH_i_ are established in wt (**d, p**), but not in *grk* (**j**_**1**_**, v**). Some *grk* S10B follicles showed a transversal V_mem_-gradient (**j**_**2**_), but such a gradient was never observed during later stages (**k,l**), in contrast to wt (**e,f**). For variability of follicles in S9 and S10B, see Additional file: Fig. S1; for numbers of analysed follicles, see Additional file: Table S3

Almost all follicles produced by transheterozygous *grk* females show morphological defects concerning both axes (Fig. 1). These *grk* follicles are characterised by a transversally uniform, cuboidal FE covering the Ooc, and by an ON located, predominantly, at the posterior end. This contrasts with wt follicles, where the dorsal side in S10B is defined by a thicker, columnar FE and an anterodorsal position of the ON [11]. These morphological peculiarities correlate with stage-specific differences between wt and *grk* concerning V_mem_- and pH_i_-patterns in the FE*,* as revealed by the potentiometric dye DiBAC and the pH-indicator CFDA, respectively (Fig. 2). As described earlier [4, 7], stronger fluorescence intensities refer to more depolarised V_mem_ or more alkaline pH_i_, whereas weaker fluorescence intensities refer to more hyperpolarised V_mem_ or more acidic pH_i_.

During early to mid-vitellogenic stages S8-10A, overall V_mem_- and pH_i_-patterns of wt (Fig. 2a-c and m-o) and *grk* (Fig. 2g-i and s-u) are rather similar, since d-v gradients have not yet emerged in the wt (cf. [4, 7]). In both wt and *grk,* the somatic FE in S8 is more depolarised and more acidic than the germline cells (Ooc and NC). During S9-10A, *grk* follicles develop a similar a-p V_mem_-pattern as wt follicles, the mainbody follicle cells (mFC) being hyperpolarised in relation to the neighbouring pFC and centripetal follicle cells (cFC). In addition, during S9-10A, a-p pH_i_-gradients are present in the FE of both wt and *grk*, the pFC being most alkaline. Additionally, in both genotypes, the anterior-most NC is the most alkaline.

**Fig. 3:**
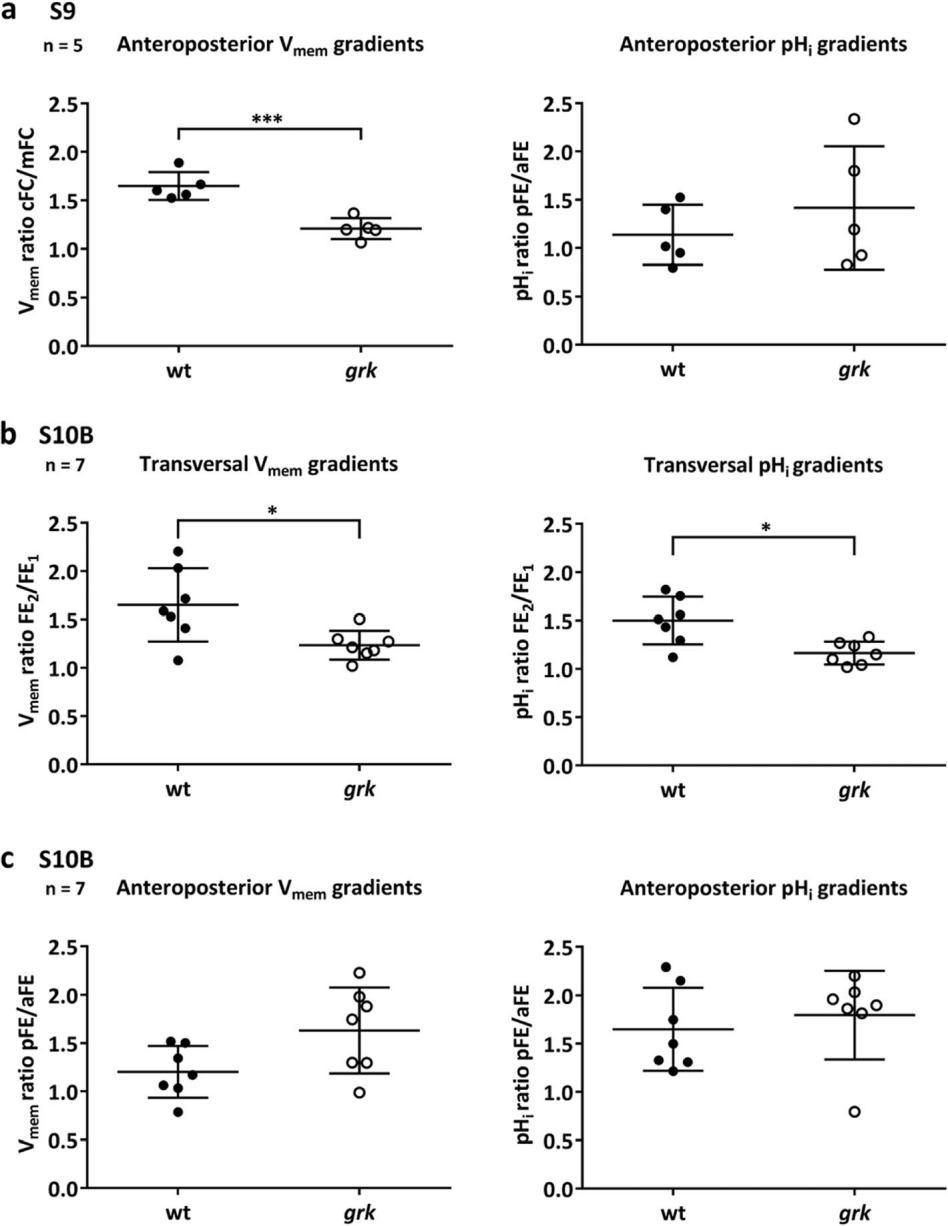
Compared to wt, in *grk* S9, the a-p V_mem_-gradient is significantly shallower, and in *grk* S10B, transversal V_mem_- and pH_i_-gradients are missing in the FE. **a** The a-p V_mem_-gradients (fluorescence intensity ratios cFC/mFC) in S9 were evaluated as described previously [7]. The a-p pH_i_-gradients (fluorescence intensity ratios pFE/aFE) were quantified according to Fig. 1e_2_. The higher the fluorescence intensity ratio is, the steeper is the gradient. Mean values (± SD, standard deviation) were compared using an unpaired t-test. Difference for V_mem_-gradients is significant (*** *p* < 0.001); difference for pH_i_-gradients is not significant (pH_i_-gradients are shallower than V_mem_-gradients). **b** Transversal gradients (fluorescence intensity ratios FE_2_/FE_1_; larger value vs. smaller value), and **c** a-p gradients (fluorescence intensity ratios pFE/aFE) in S10B were quantified as shown in Fig. 1e. Differences for transversal gradients are significant (* *p* < 0.05); differences for a-p gradients are not significant

However, in the S9 FE of wt and *grk*, a closer look reveals that the slopes of the a-p V_mem_-gradients differ (Fig. 2b and h; for variability between follicles of the same stage, see Additional file: Fig. S1). Compared to wt, the mFC in *grk* are less hyperpolarised in relation to neighbouring cFC, and the whole FE is more depolarised, resulting in a shallower a-p V_mem_-gradient (significantly reduced angle of gradient; Table 1 and Fig. 3a).

**Table 1:** In the S9 FE of *grk*, the a-p V_mem_-gradient is shallower

	**V**_**mem**_	**pH**_**i**_
**Gradient**	**Fraction of S9 follicles**	**Fraction of S9 follicles**
**a-p**	**with cFC/mFC ≥ 1.5**^**§**^	**with pFE/aFE ≥ 1.3**^**#**^
wt	5/5	2/5
*grk*	0/5	2/5

^§^a-p V_mem_-gradients (fluorescence intensity ratios cFC/mFC) in the S9 FE of wt and *grk* were evaluated as described previously [7]. ^#^a-p pH_i_-gradients (fluorescence intensity ratios pFE/aFE) were quantified according to Fig. 1e_2_. The higher the fluorescence intensity ratio is, the steeper is the gradient (*n* = 5; see also Fig. 3a)

Even more striking differences become obvious during S10B, when d-v electrochemical gradients are established in the FE of wt (Fig. 2d and p) but not *grk* (Fig. 2j_1_ and v; Table 2 and Fig. 3b; for variability between follicles of the same stage, see Additional file: Fig. S1). In most analysed wt S10B follicles, according to the position of the ON, the more depolarised or more alkaline side was identified as the ventral side (cf. [4, 7]). In some *grk* S10B follicles, a transversal V_mem_-gradient was detected (Fig. 2j_2_; Additional file: Table S1) but, in contrast to wt, such a gradient was absent from later stages (S11–12; Fig. 2e,f and k,l). A transversal pH_i_-gradient, however, was never observed in *grk* (Fig. 2v-x; Table 2 and Fig. 3b; Additional file: Table S1). Concerning a-p electrochemical gradients, significant differences between wt and grk were not observed (Table 2 and Fig. 3c; Additional file: Table S2).

**Table 2:** In the S10B FE of *grk*, distinct transversal V_mem_- and pH_i_-gradients are missing

	**V**_**mem**_	**pH**_**i**_
**Gradients**	**Fraction of S10B follicles**	**Fraction of S10B follicles**
**transversal**^**§**^	**with FE**_**2**_**/FE**_**1**_ **≥ 1.5**	**with FE**_**2**_**/FE**_**1**_ **≥ 1.5**
wt	5/7	4/7
*grk*	1/7	0/7
**a-p**^**#**^	**with pFE/aFE ≥ 1.5**	**with pFE/aFE ≥ 1.5**
wt	2/7	4/7
*grk*	4/7	6/7

^§^Transversal gradients (fluorescence intensity ratios FE_2_/FE_1_; larger value vs. smaller value), and ^#^a-p gradients (fluorescence intensity ratios pFE/aFE) in the S10B FE of wt and *grk* were quantified as shown in Fig. 1e (for variability see Additional file: Tables S1 and S2). The higher the fluorescence intensity ratio is, the steeper is the gradient (*n* = 7; see als Fig. 3b and c). Since, in *grk*, the cFC and mFC (refering to aFE) are often both more hyperpolarised and more acidic than the aberrant pFC, a-p gradients in *grk* seem to be somewhat steeper than in wt (cf. Additional file: Fig. S1 and Table S2), but this difference was not significant (cf. Fig. 3c)

### Cytoskeletal differences between wt and *grk*

**Fig. 4:**
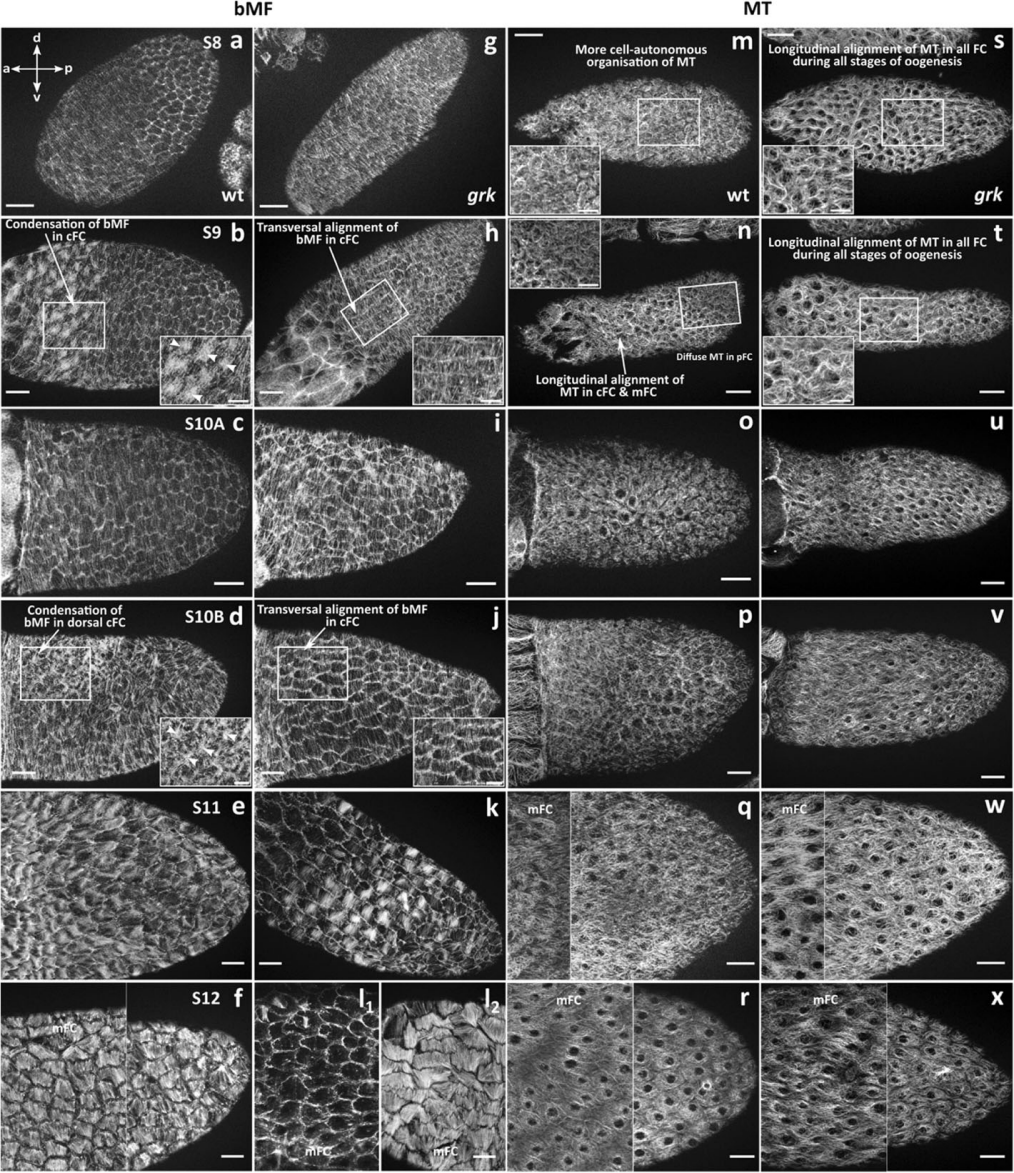
Compared to wt, the *grk* FE exhibits striking cytoskeletal differences. Staining of bMF using fluorescent phalloidin (**a-l**), and staining of MT using an antibody against acetylated α-tubulin (**m-x**); tangential optical sections (SIM) of typical S8–12 follicles. **a-l** Fluorescent phalloidin (bMF) stained wt (**a-f**) and *grk* (**g-l**) follicles. **m-x** Anti-tubulin (MT) stained wt (**m-r**) and *grk* (**s-x**) follicles (scale bars respresent 20 μm; composed micrographs show different regions of the same follicle, except for (**l**). The *grk* FE shows prominent pecularities concerning the bMF-pattern: In contrast to wt, bMF in *grk* are characterised by strict transversal alignment during all analysed stages (**g-l**), while wt-typical condensations of bMF in cFC (S9, **b**) and dorsal cFC (S10B, **d**) are absent. However, in *grk* S11, many FC show no bMF (**k**). In several *grk* S12 follicles, bMF were almost totally missing (**l**_**1**_), while other follicles (**l**_**2**_) exhibit transversally aligned bMF which are less strictly transcellularly organised than in wt. On the other hand, we observed strict longitudinal MT-alignment in the whole *grk* FE during all analysed stages (**s-x**), whereas in the wt FE, longitudinal MT-alignment continuously expands from cFC (S9, **n**) to pFC (S12, **r**). For variability of follicles in S9 and S10B, see Additional file: Fig. S2; for numbers of analysed follicles, see Additional file: Table S3

**Fig. 5:**
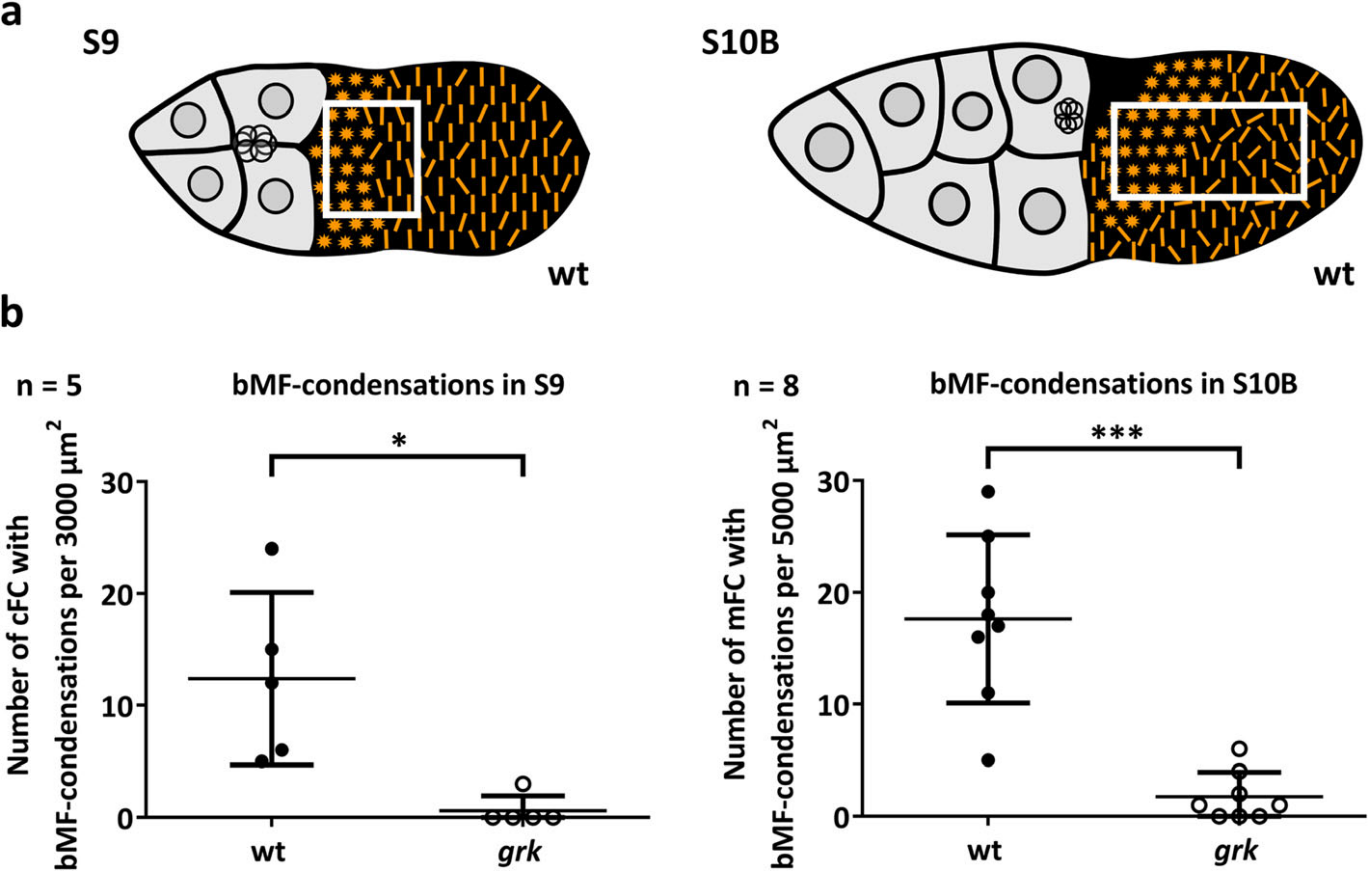
Comparison of wt and *grk* concerning condensations of bMF in S9 (cFC) and S10B (mFC). Wt-typical condensations (for regions of interest, see boxes marked in **a**) are missing in *grk* (**b**). Mean values (± SD, standard deviation) were compared using an unpaired t-test (* *p* < 0.05; *** *p* < 0.001)

Using fluorescent phalloidin and an antibody against acetylated α-tubulin, we compared, during S8–12, the FE of wt and *grk* concerning cytoskeletal organisation (Figs. 4 and 5; for wt, cf. [8]). During S8, basal microfilaments (bMF) show the same parallel transversal alignment in wt and *grk* (Fig. 4a,g). Except for wt S10A (Fig. 4c), this alignment is missing in wt S9, S10B and S11, but it persists during these stages in *grk*. In all cFC in wt S9, and in dorsal cFC in wt S10B, condensations of bMF appear (Fig. 5; cf. [8]). This phenomenon is accompagnied by a loss of the transcellular parallel alignment of bMF in the remaining wt FE (Fig. 4b, d; for variability between follicles of the same stage, see Additional file: Fig. S2). In wt S11, a rearrangement of bMF occurs with fan-shaped structures (Fig. 4e; cf. [8]), and in wt S12, a dense transversal pattern of parallel bMF develops (Fig. 4f). In *grk* S11*,* many FC seem to contain no bMF (Fig. 4k). Moreover, in several *grk* S12 follicles, bMF were almost totally missing, while other follicles exhibit transversally aligned bMF (Fig. 4l). Thus, as observed for bioelectrical properties, the *grk* FE exhibits striking stage-specific pecularities concerning the bMF-pattern. In contrast to wt, *grk* bMF are characterised by transversal alignment during all analysed stages, while wt-typical condensations in cFC (S9) and dorsal cFC (S10B) are absent (Fig. 4g-l; Fig. 5).

During S8, all wt FC show a more or less cell-autonomous organisation of MT, being arranged around the nuclei (Fig. 4m). Beginning with S9, the MT in wt cFC and mFC develop a longitudinal alignment, while the MT in pFC maintain their cell-autonomous arrangement (Fig. 4n). The longitudinal MT-alignment begins in cFC and spreads out over mFC to pFC (cf. [8]). However, in *grk*, in the whole FE, longitudinal alignment of MT was observed during all analysed stages (Fig. 4s-x). Between neighbouring FC, this MT-alignment in *grk* appears to be more coordinated than in wt. Thus, as observed for bMF, the wt MT-pattern (Fig. 4m-r) is more cell-autonomously organised, whereas the *grk* MT-pattern is characterised by a coordinated transcellular organisation along the longitudinal axis (Fig. 4s-x).

**Fig. 6:**
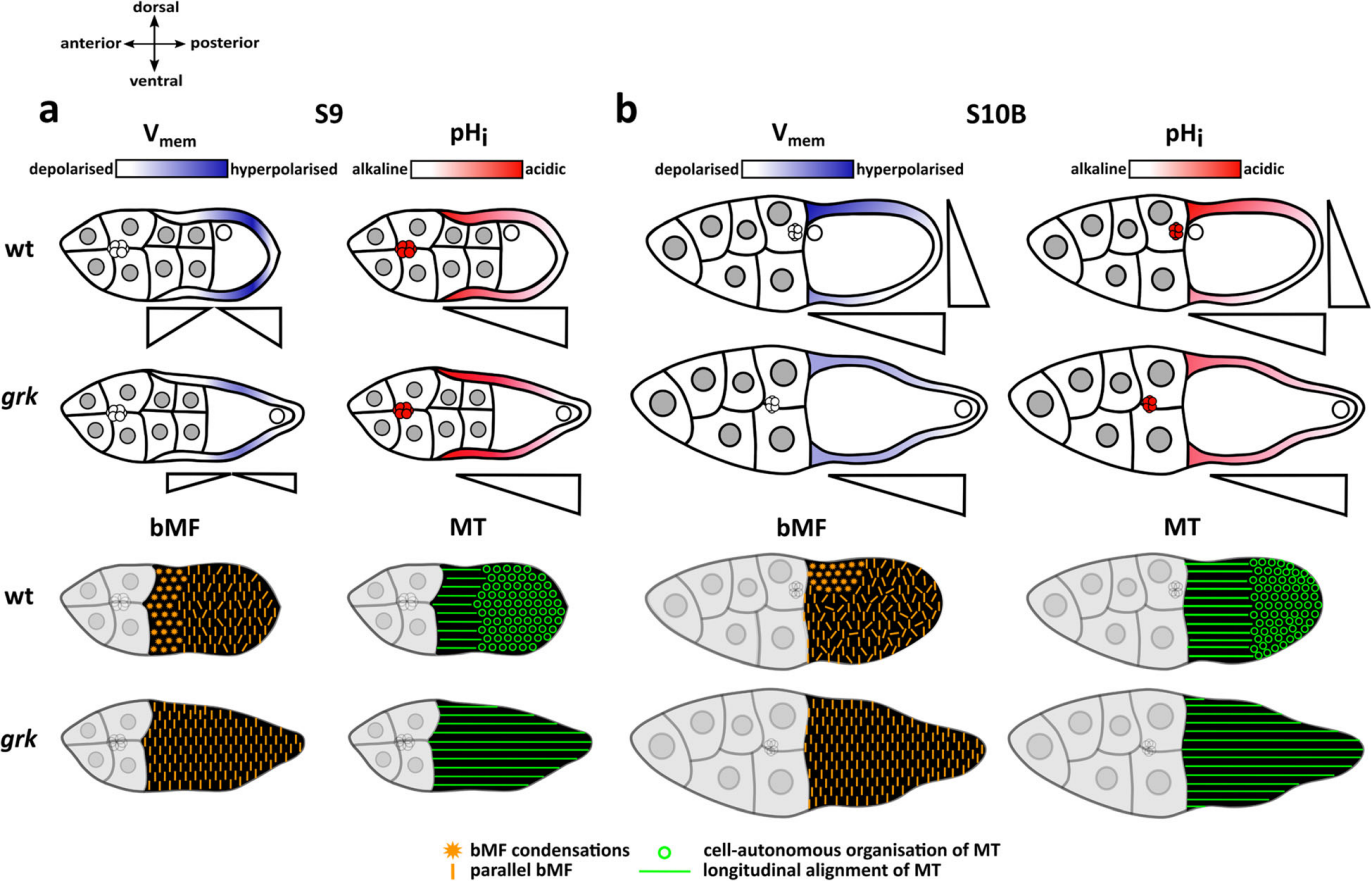
Summary of prominent bioelectrical and cytoskeletal differences between the FE of wt and *grk*. In early vitellogenic stages, as for example S9 (**a**), patterns of V_mem_ (white to blue gradient) and pH_i_ (white to red gradient) in *grk* are similar to the respective patterns in wt. White refers to stronger fluorescence intensities, corresponding to relative depolarisation or relative alkalisation, while blue (V_mem_) and red (pH_i_) refer to weaker fluorescence intensities, corresponding to relative hyperpolarisation (blue) or relative acidification (red). Triangles indicate fluorescence-intensity gradients. In S9, both wt and *grk* show a-p V_mem_-gradients, with mFC being hyperpolarised (blue) in relation to neighbouring cFC and pFC (white). The same holds true for a-p pH_i_-gradients, with pFC being the most alkaline FC (white). However, the a-p V_mem_-gradient in *grk* is shallower, since mFC are less hyperpolarised relative to neighbouring FC, and the whole FE is more depolarised compared to wt. In S10B (**b**), in relation to other FC, *grk* cFC show both slight hyperpolarisation and slight acidification, while dorsal wt cFC show strong hyperpolarisation as well as strong acidification. In both wt and *grk,* the BC exhibit relatively depolarised V_mem_ and relatively acidic pH_i_. Concerning cytoskeletal organisation in S9 (**a**), *grk* bMF are characterised by transversal alignment (orange dashes) and *grk* MT by longitudinal alignment (green lines), while wt-typical bMF-condensations (orange asterisks) and cell-autonomously organised MT (green circles) are absent. From S10B onward (**b**), prominent d-v gradients of both V_mem_ and pH_i_ appear in the FE of wt, but not *grk*. This corresponds with peculiarities in both the bMF- and the MT-organisation: In the wt FE, condensations of bMF (orange asterisks) appear in dorsal cFC, and cell-autonomously organised MT (green circles) appear in mFC and pFC. In contrast, in *grk*, both condensations of bMF and cell-autonomously organised MT are absent from the whole FE

The characteristic bioelectrical and cytoskeletal features of wt and *grk* in S9 and S10B are summarised in Fig. 6. In early vitellogenic stages (up to S9), a-p gradients of V_mem_ and pH_i_ show the same polarity in both genotypes. However, the a-p V_mem_-gradient in *grk* S9 is shallower, and the whole FE is more depolarised compared to wt. In *grk* S9, bMF are characterised by transversal alignment and MT by longitudinal alignment, whereas both wt-typical condensations of bMF and cell-autonomously organised MT are absent. During S10B, striking bioelectrical as well as cytoskeletal differences appear, when d-v polarity becomes obvious in wt but not in *grk*. In wt S10B, prominent a-p and d-v gradients of both V_mem_ and pH_i_ appear in combination with condensations of bMF in dorsal cFC and cell-autonomously organised MT in mFC and pFC. In *grk* S10B, however, d-v V_mem_- and pH_i_-gradients are missing and both condensations of bMF and cell-autonomously organised MT are absent (Fig. 6).

## Discussion

### Altered axial polarity correlates with altered electrochemical gradients

At the posterior pole of *grk* follicles older than S9, migrating FC can be observed which, more or less, enclose the ON. According to previous reports [9, 17], the three anterior FC types (BC, stretched FC and cFC) are duplicated at the posterior end of *grk* follicles. The “posterior FC” in *grk* undergo similar morphological movements as the anterior FC. “Posterior BC” lose their epithelial organisation, “adjacent posterior FC” become stretched and “posterior cFC” migrate centripetally, sometimes even bisecting the Ooc. These aberrations of axial polarity in the FE of *grk* correlate with altered bioelectrical and cytoskeletal patterns as described in the present study.

In the FE of wt and *grk*, we compared stage-specific longitudinal and transversal gradients of V_mem_ and pH_i_, respectively. Since d-v electrochemical gradients are not yet established in the wt during early to mid-vitellogenic stages S8-10A (cf. [4, 7]), the overall V_mem_- and pH_i_-patterns of wt and *grk* are rather similar. However, corresponding to impaired a-p polarity in *grk*, the slope of the a-p V_mem_-gradient in S9 is significantly reduced compared to wt*.*

More striking bioelectrical characteristics relating to missing d-v polarity in *grk* appear in S10B. During *grk* S10B-12, significant transversal electrochemical gradients are absent. In early wt S10B, the FE becomes continuously depolarised from dorsal to ventral while pH_i_ increases in the same direction. During late wt S10B-12, dorsal cFC show increasing depolarisation (cf. [7]). Refering to morphological variability, some *grk* S10B follicles exhibit a transient transversal V_mem_-gradient which was never observed during later stages.

### Altered electrochemical gradients correlate with altered cytoskeletal patterns

As shown in detail recently [8], stage-specific alterations of V_mem_ and pH_i_ correlate with structural modifications of bMF and MT in the wt FE. Higher pH_i_, as observed in mFC and pFC in S10B, stabilises the parallel alignment of bMF and results in loss of the longitudinal alignment of MT (leading to a more cell-autonomous MT-arrangement). Lower pH_i_, as observed in dorsal cFC in early S10B, leads to increasing disorder and condensation of bMF as well as to stabilisation of the longitudinal MT-alignment. Lower pH_i_ in combination with relatively depolarised V_mem_, as observed in dorsal cFC in late S10B, contributes to the disintegration of bMF. Correlations between bioelectrical properties and cytoskeletal patterns, as observed in different stages and different regions of the wt FE, correspond to correlations induced by inhibitors of various ion-transport mechanisms [8]. These observations lend support to the hypothesis that gradual modifications of electrochemical signals can serve as physiological means to regulate cell and tissue architecture by modifying cytoskeletal patterns [7, 8].

Further support to this hypothesis is provided by the present study. Shallower (or no) V_mem_-gradients and relative alkalisation, as generated by the inhibition of certain ion-transport mechanisms [7], lead to stabilisation of the parallel transversal bMF-pattern in wt [8]. This also holds true for the cFC in *grk* S9 and the dorsal cFC in *grk* S10B, where bMF retain their transversal alignment while wt-typical condensation and subsequent disintegration of bMF are missing. Similarly, shallower (or no) V_mem_-gradients lead to stabilisation of the longitudinal MT-orientation in wt [8]. This also holds true for mFC and pFC in *grk* S9 as well as in *grk* S10B, where, in addition, a transversal pH_i_-gradient is missing. In the whole *grk* FE*,* MT exhibit a longitudinal transcellular alignment, whereas in wt mFC and pFC, MT-patterns are characterised by a more cell-autonomous organisation.

In *grk,* preferential alignment of bMF along the transversal axis and of MT along the longitudinal axis is obviously enhanced. Assuming a duplication of anterior FC types at the “posterior pole” in *grk* [9, 14, 17], it seems plausible that both the transversal alignment of bMF and the longitudinal alignment of MT, as found in the anterior FE of *grk*, is duplicated in the “posterior FE”.

### Bioelectrical and cytoskeletal polarity depend on axial polarity

Considering the described wt- and *grk*-specific bioelectrical and cytoskeletal features (for summary, see Fig. 6), it is obvious that bioelectrical and cytoskeletal polarity are linked to axial polarity. The establishment of electrochemical gradients in the FE depends on asymmetrically distributed or activated ion-transport mechanisms and gap junctions [4, 7, 40–44]. This asymmetry is presumed to depend on early Grk-Egfr signalling and continues to exert influence on cytoskeletal patterns later in development [7, 8].

In wt S9, longitudinal electrochemical gradients with relative depolarisation and relative acidification in cFC result in condensation of bMF and in longitudinally aligned MT in this area [8]. Impaired a-p polarity in *grk* S9, however, leads to relative depolarisation in the whole FE resulting in a shallower longitudinal V_mem_-gradient and in stabilisation of the transversal bMF-pattern. In wt S10B, strong transversal electrochemical gradients, showing relative hyperpolarisation and relative acidification in dorsal cFC, lead to condensation and disintegration of bMF [8]. On the other hand, as a consequence of missing d-v polarity in *grk* S10B, transversal electrochemical gradients as well as bMF-condensation and disintegration are absent from the whole FE.

Therefore, we propose that shallow or missing electrochemical gradients, as observed in *grk,* result in stabilisation of cytoskeletal patterns. Throughout oogenesis, the bMF remain oriented along the transversal axis while the MT remain oriented along the longitudinal axis. This interpretation corresponds to the previous observation that experimentally reduced V_mem_-gradients stabilise both bMF- and MT-patterns [8].

## Conclusion

Our analysis of the *Drosophila* mutant *grk* leads to the conclusion that not only cell-specific levels of V_mem_ and pH_i_, or the polarities of electrochemical gradients [8], but also the slopes of these gradients are crucial for either alteration or stability of cytoskeletal patterns. When primary signals of axial polarity, like Grk, are weak or missing, ion-transport mechanisms and gap junctions in the FE are not distributed or activated asymmetrically. Consequently, electrochemical gradients are shallow and patterns of cytoskeletal elements remain unchanged.

## Methods

### Fly stocks

For analysis, *Drosophila melanogaster* wild-type Oregon R (wt) and *gurken* (*grk*) were used. The strains *w; grk*^*HF48*^*/CyO* and *w; grk*^*2B6*^*/CyO* (gift of S. Roth and O. Karst, Köln, Germany) were crossed to generate transheterozygous *grk*^*HF48*^*/grk*^*2B6*^ flies. Although *grk*^*2B6*^ is the strongest existing allele [14, 45, 46], only a combination of both *grk* null alleles led to a penetrance of 100% ventralised *grk* follicles (Fig. 1c). Flies were reared at 25 °C in the dark on standard food with additional fresh yeast.

### Preparation of follicles

Females were killed by crushing the head with tweezers without anaesthesia, and 3 days old wt or 2 days old *grk* ovaries were dissected (older *grk* ovaries contained many degenerating follicles). Single follicles of stages S8–12 were isolated from the epithelial sheath by pulling at the anterior tip of an ovariole. Dissection was carried out in *Drosophila* phosphate-buffered saline [47]. For staining with fluorescent indicators, we used R-14 Medium [47] which is best suited for in-vitro culture of *Drosophila* follicles [48].

### Fluorescent membrane potential indicator

For the analysis of V_mem_-patterns, we used the fluorescent potentiometric dye DiBAC (DiBAC_4_(3); bis-(1,3-dibutylbarbituric acid) trimethine oxonol; Molecular Probes/Thermo Fisher Scientific, USA). As described earlier [4, 7], relative depolarisation leads to intracellular accumulation of the anionic dye and to increasing fluorescence while relative hyperpolarisation leads to decreasing fluorescence. Living follicles were incubated for 20 min in R-14 medium containing 4 μM DiBAC (dissolved in 70% ethanol). Thereafter, they were mounted in R-14 medium and analysed immediately using × 10/0.25 and × 20/0.5 objectives and median optical sections (Fig. 1d) on a Zeiss AxioImager.M2 structured-illumination microscope (SIM), equipped with a Zeiss ApoTome, a Zeiss AxioCamMRm camera and the appropriate filter set. For numbers of analysed S8–12 follicles, see Additional file: Table S3.

### Fluorescent intracellular pH indicator

For the analysis of pH_i_-patterns, we used the fluorescent pH-indicator CFDA (5-CFDA,AM; 5-carboxyfluorescein diacetate, acetoxymethyl ester; Molecular Probes) which enters cells as an anion. As described earlier [4, 7], increasing fluorescence indicates relative alkalisation while decreasing fluorescence due to protonation indicates relative acidification. Living follicles were incubated for 20 min in R-14 medium containing 4 μM CFDA (dissolved in dimethyl sulfoxide, DMSO). Subsequently, the follicles were mounted in R-14 medium and viewed immediately as described above using median optical sections (Fig. 1d). For numbers of analysed S8–12 follicles, see Additional file: Table S3.

### F-actin staining using fluorescent phalloidin

Follicles were fixed in microfilament-stabilising buffer (MF-buffer [8, 38]) with 4% formaldehyde and 0.2% Triton X-100 for 20 min at room temperature, washed with phosphate-buffered saline (PBS) and stained with 0.25 μg/ml phalloidin-FluoProbes 550A (Interchim, France; dissolved in DMSO) in PBS. After washing, the follicles were mounted in Fluoromount G (Interchim) and viewed as described above using a × 40/1.3 oil objective and tangential optical sections (Fig. 1d). For numbers of analysed S8–12 follicles, see Additional file: Table S3.

### Indirect immunofluorescence staining of microtubules

Follicles were fixed for 20 min at room temperature in MF-buffer as described above, washed with PBS containing 0.1% Triton X-100 and blocked for 1 h at room temperature with 2% bovine serum albumin (BSA)/0.1% Triton X-100 in PBS. Thereafter, the follicles were incubated overnight at 4 °C or for 1 h at room temperature in PBS containing 1% BSA/0.1% Triton X-100 and a monoclonal antibody against acetylated α-tubulin (6-11B-1; Santa Cruz Biotechnology, USA) diluted 1:100 [8]. After washing, the follicles were treated for 1 h at room temperature with goat anti-mouse-biotin (Dianova, Germany) diluted 1:200 in PBS containing 1% BSA/0.1% Triton X-100. Washing was repeated before the follicles were incubated for 30 min with streptavidin-TexasRed (Dianova) diluted 1:100 in PBS containing 1% BSA/0.1% Triton X-100. After washing, the follicles were mounted and analysed as described above using tangential optical sections (Fig. 1d). For numbers of analysed S8–12 follicles, see Additional file: Table S3. Controls were performed without primary antibody.

### Staging of follicles and determination of axes

Follicles were staged according to criteria described previously [11, 49]. To determine the a-p axis, the anterior position of the NC was used as marker, while for the d-v axis, the anterodorsal position of the ON and the columnar dorsal FE (S10B) were used. For *grk* follicles, the same criteria for staging and axis determination were applied. Due to the posterior location of the ON and the transversally homogeneous FE, no dorsal side was detectable in *grk* (Fig. 1).

### Quantification of fluorescence intensities in the FE

To quantify both the longitudinal and the transversal V_mem_- and pH_i_-gradients in the FE of *grk* and wt, respectively, we used median optical sections (Fig. 1d) of stained follicles. Fluorescence itensities (“mean grey value”) of both sides (FE_1_ and FE_2_ or aFE and pFE) were measured using ImageJ (Fig. 1e) and a ratio of both values was determined. For a-p V_mem_-gradients in S9, fluorescence intensities of cFC, mFC and pFC were measured separately and ratios determined according to [7].

## Supplementary information

**Additional file 1: Figure S1.** Typical dorsoventral electrochemical gradients, as observed in the wt FE beginning with S10B, are absent in *grk*. Additional examples corresponding to Fig. 2, showing the variability between follicles of the same stage. **Table S1.** Quantification of fluorescence intensities of transversal electrochemical gradients in the FE of wt and *grk* (S10B). Data corresponding to Table 2 and Fig. 3b. **Table S2.** Quantification of fluorescence intensities of anteroposterior electrochemical gradients in the FE of wt and *grk* (S10B). Data corresponding to Table 2 and Fig. 3c. **Figure S2.** The *grk* FE exhibits striking cytoskeletal differences compared to wt (S9 and S10B). Additional examples corresponding to Fig. 4, showing the variability between follicles of the same stage. **Table S3**. Numbers of follicles analysed for each condition and developmental stage.

## Data Availability

The datasets used during the current study are available from the corresponding author on reasonable request.

## Abbreviations

5-CFDA,AM: 5-Carboxyfluorescein diacetate, acetoxymethyl ester
aFE: Anterior half of the FE
a-p: Anteroposterior
BC: Border cells
bMF: Basal microfilaments
BSA: Bovine serum albumine
cFC: Centripetal follicle cells
DiBAC_4_(3): Bis-(1,3-dibutylbarbituric acid) trimethine oxonol
DMSO: Dimethyl sulfoxide
d-v: Dorsoventral
FC: Follicle cells
FE: Follicular epithelium
*grk*: *gurken*
MF: Microfilaments
mFC: Mainbody follicle cells
MT: Microtubules
NC: Nurse cells
ON: Oocyte nucleus
Ooc: Oocyte
PBS: Phosphate-buffered saline
pFC: Posterior follicle cells
pFE: Posterior half of the FE
pH_i_: Intracellular pH
S: Stage
SIM: Structured-illumination microscopy
V_mem_: Membrane potential
wt: Wild-type
