# Supplementary material for: Bioelectrical and cytoskeletal patterns correlate with altered axial polarity in the follicular epithelium of the Drosophila mutant gurken
Source: BMC Dev Biol. 2020 Mar 13;20:5. doi: 10.1186/s12861-020-00210-8 (PMC7071586; doi:10.1186/s12861-020-00210-8)
Supplement: Supplementary file 1 — Additional file 1: Figure S1. Typical dorsoventral electrochemical gradients, as observed in the wt FE beginning with S10B, are absent in grk. Additional examples corresponding to Fig. 2, showing the variability between follicles of the same stage. Table S1. Quantification of fluorescence intensities of transversal electrochemical gradients in the FE of wt and grk (S10B). Data corresponding to Table 2 and Fig. 3b. Table S2. Quantification of fluorescence intensities of anteroposterior electrochemical gradients in the FE of wt and grk (S10B). Data corresponding to Table 2 and Fig. 3c. Figure S2. The grk FE exhibits striking cytoskeletal differences compared to wt (S9 and S10B). Additional examples corresponding to Fig. 4, showing the variability between follicles of the same stage. Table S3. Numbers of follicles analysed for each condition and developmental stage. [file 12861_2020_210_MOESM1_ESM.pdf]

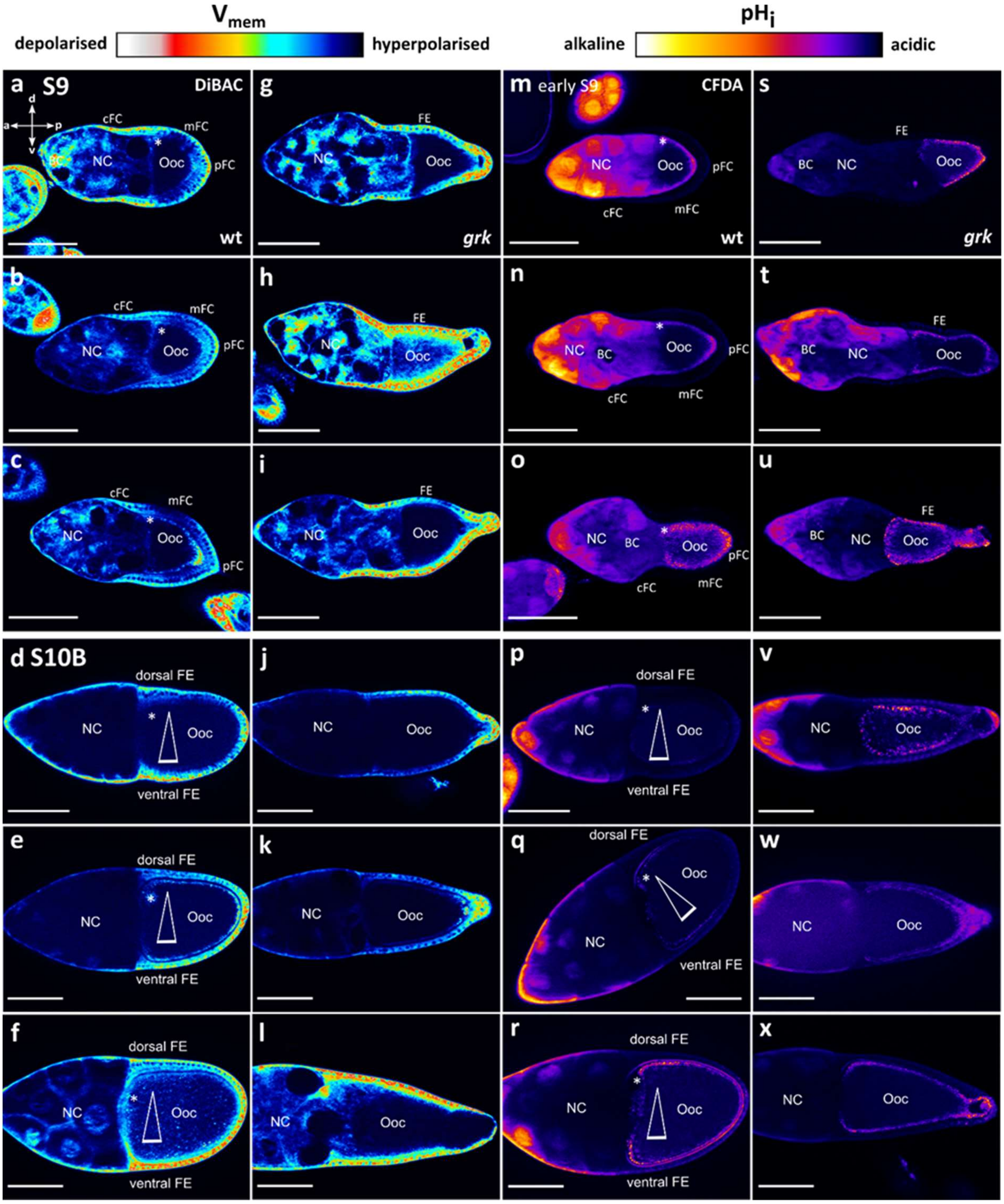

Figure S1: Typical dorsoventral electrochemical gradients, as observed in the wt FE beginning with S10B, are absent in *grk*. Additional examples corresponding to Fig. 2, showing the variability between follicles of the same stage. **a-l:** Pseudocolor images of DiBAC (V_{mem}) stained wt S9 (**a-c**) and wt S10B (**d-f**) as well as *grk* S9 (**g-i**) and *grk* S10B (**j-l**), respectively. **m-x:** Pseudocolor images of CFDA (pH_i) stained wt S9 (**m-o**) and wt S10B (**p-r**) as well as *grk* S9 (**s-u**) and *grk* S10B (**v-x**), respectively (scale bars represent 100 µm; triangles indicate gradient's polarity; positions of the ON are marked with asterisks)

Table S1: Quantification of fluorescence intensities of transversal electrochemical gradients in the FE of wt and *grk* (S10B).

Mean fluorescence intensity in the FE of S10B follicles§		wt FE_1	wt FE_2	wt ratio	*grk* FE_1	*grk* FE_2	*grk* ratio
V_{mem}	**follicle 1**	47.549	96.665	2.03	53.940	81.147	1.50
	**follicle 2**	87.134	93.925	1.08	54.312	64.269	1.18
	**follicle 3**	50.875	77.874	1.53	116.148	141.018	1.21
	**follicle 4**	54.227	86.196	1.59	91.955	119.399	1.30
	**follicle 5**	66.310	93.636	1.41	58.158	59.392	1.02
	**follicle 6**	82.120	140.956	1.72	56.750	72.321	1.27
	**follicle 7**	44.366	97.796	2.20	28.814	33.256	1.15
pH_i	**follicle 1**	27.376	39.256	1.43	15.877	11.915	1.33
	**follicle 2**	7.963	13.977	1.76	1.091	1.354	1.24
	**follicle 3**	5.782	9.033	1.56	18.275	21.024	1.15
	**follicle 4**	9.352	10.494	1.12	26.991	25.883	1.04
	**follicle 5**	16.600	21.542	1.30	15.032	19.093	1.27
	**follicle 6**	8.997	13.623	1.51	11.018	10.802	1.02
	**follicle 7**	12.231	22.268	1.82	6.437	7.085	1.10

§Data corresponding to Table 2 and Fig. 3b. Fluorescence intensities ("mean grey value") of both sides of the FE (FE_1 and FE_2) were measured using ImageJ (see Fig. 1e). The ratio is FE_2/FE_1, larger value vs. smaller value. In some wt follicles which were in late S10B (numbers 3 and 7), d-v polarity of the transversal V_{mem}-gradient was reversed (dorsal side depolarised), compared to earlier S10B follicles as shown in Figs. 2 and S1 (cf. [7]).

Table S2: Quantification of fluorescence intensities of anteroposterior electrochemical gradients in the FE of wt and *grk* (S10B).

Mean fluorescence intensity in the FE of S10B follicles§		wt aFE	wt pFE	wt ratio	*grk* aFE	*grk* pFE	*grk* ratio
V_{mem}	**follicle 1**	54.687	82.225	1.50	44.455	88.138	1.98
	**follicle 2**	87.093	92.574	1.06	93.915	121.706	1.29
	**follicle 3**	55.051	74.046	1.35	40.100	75.469	1.88
	**follicle 4**	65.824	68.068	1.03	51.718	67.134	1.30
	**follicle 5**	64.369	97.711	1.52	127.958	126.426	0.99
	**follicle 6**	97.739	114.622	1.17	53.689	80.274	1.74
	**follicle 7**	71.888	56.524	0.79	20.235	45.114	2.29
pH_i	**follicle 1**	21.080	45.383	1.43	7.476	14.197	1.90
	**follicle 2**	8.238	12.342	1.76	10.512	21.395	2.04
	**follicle 3**	6.710	8.905	1.56	3.685	2.927	0.79
	**follicle 4**	9.213	11.206	1.12	17.344	32.323	1.86
	**follicle 5**	11.663	26.749	1.30	11.908	23.356	1.96
	**follicle 6**	9.389	12.291	1.51	4.407	7.998	1.81
	**follicle 7**	12.306	21.521	1.82	4.103	9.043	2.20

§Data corresponding to Table 2 and Fig. 3c. Fluorescence intensities ("mean grey value") of the anterior and posterior half of the columnar FE (aFE and pFE) were measured using ImageJ (see Fig. 1e). The ratio is pFE/aFE.

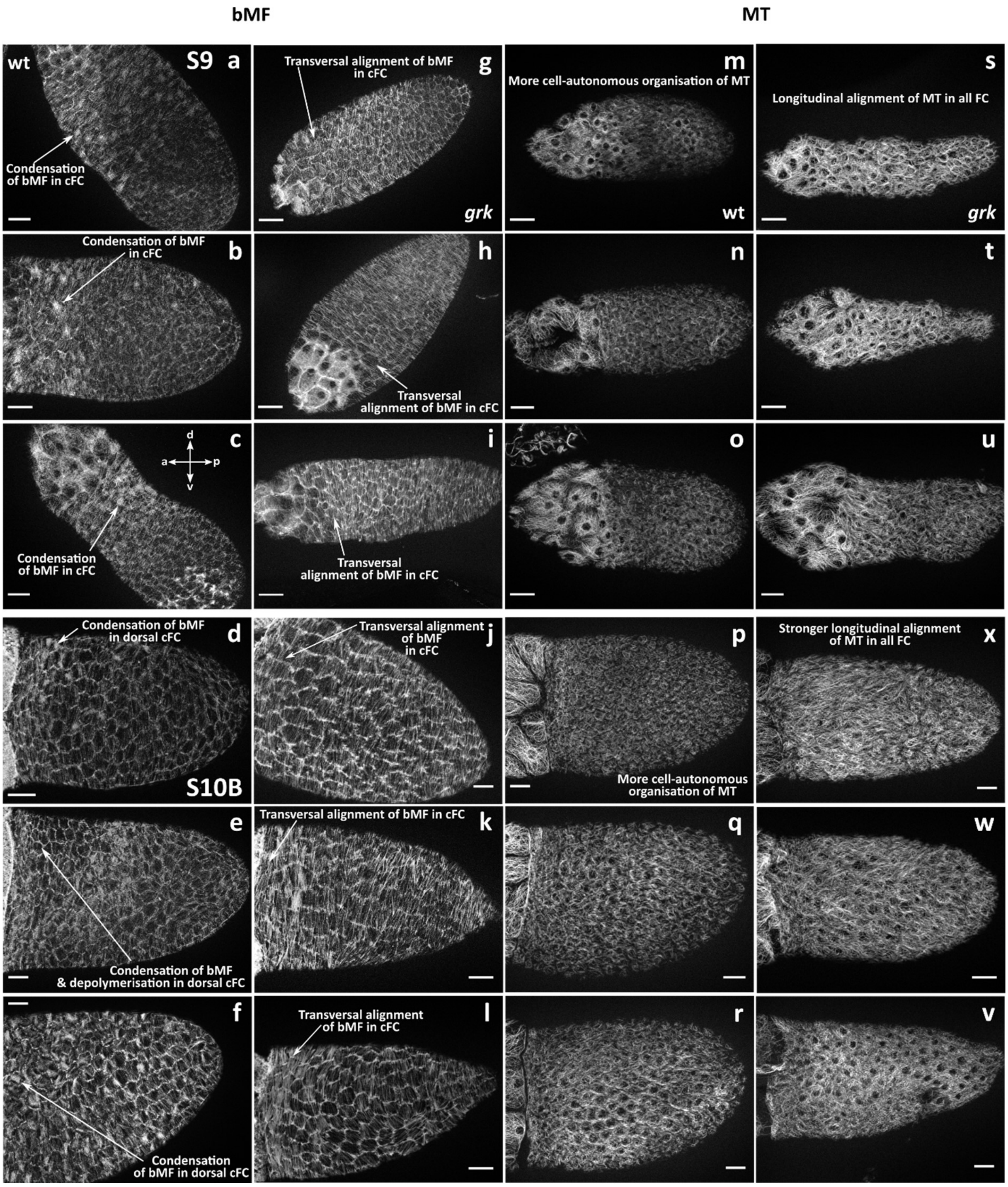

Figure S2: The *grk* FE exhibits striking cytoskeletal differences compared to wt (S9 and S10B). Additional examples corresponding to Fig. 4, showing the variability between follicles of the same stage. **a-l:** Fluorescent phalloidin (bMF) stained wt S9 (**a-c**) and wt S10B (**d-f**) as well as *grk* S9 (**g-i**) and *grk* S10B (**j-l**), respectively. **m-x:** Antitubulin (MT) stained wt S9 (**m-o**) and wt S10B (**p-r**) as well as *grk* S9 (**s-u**) and *grk* S10B (**v-x**), respectively (scale bars respresent 20 µm).

Table S3: Numbers (n) of follicles analysed for each condition and developmental stage.

**wt**				**grk**			
**V_{mem}**	**n**	**pH_i**	**n**	**V_{mem}**	**n**	**pH_i**	**n**
**S8**	27	**S8**	18	**S8**	9	**S8**	13
**S9**	15	**S9**	19	**S9**	24	**S9**	24
**S10A**	19	**S10A**	13	**S10A**	13	**S10A**	5
**S10B**	41	**S10B**	23	**S10B**	9	**S10B**	15
**S11**	12	**S11**	19	**S11**	6	**S11**	6
**S12**	10	**S12**	12	**S12**	5	**S12**	8
**bMF**	**n**	**MT**	**n**	**bMF**	**n**	**MT**	n
**S8**	5	**S8**	18	**S8**	11	**S8**	9
**S9**	7	**S9**	15	**S9**	26	**S9**	17
**S10A**	5	**S10A**	13	**S10A**	12	**S10A**	6
**S10B**	16	**S10B**	22	**S10B**	13	**S10B**	8
**S11**	7	**S11**	9	**S11**	8	**S11**	4
**S12**	9	**S12**	16	**S12**	11	**S12**	7
